# The effect of a combined sprint training intervention on sprint force-velocity characteristics in junior Australian football players

**DOI:** 10.7717/peerj.14873

**Published:** 2023-03-15

**Authors:** Dylan Shaun Hicks, Claire Drummond, Kym J. Williams, Roland van den Tillaar

**Affiliations:** 1 SHAPE Research Centre, Flinders University of South Australia, Bedford Park, South Australia, Australia; 2 Sport Science & Physical Education, Nord University, Levanger, Norway

**Keywords:** Acceleration, Sprinting, Supramaximal, Power, Biomechanics, Force-velocity profile

## Abstract

**Background:**

Sprint performance in junior Australian football (AF) players has been shown to be a differentiating quality in ability level therefore developing sprint characteristics via sprint-specific training methods is an important aspect of their physical development. Assisted sprint training is one training method used to enhance sprint performance yet limited information exists on its effect on sprint force-velocity characteristics. Therefore, the main aim of this study was to determine the influence of a combined sprint training intervention using assisted and maximal sprint training methods on mechanical characteristics and sprint performance in junior Australian football players.

**Methods:**

Upon completing familiarization and pre-testing, twenty-two male junior Australian football (AF) players (age 14.4 ± 0.3 years, body mass 58.5 ± 10.0 kg, and height 1.74 ± 0.08 m) were divided into a combined sprint training (CST) group (n = 14), and a maximal sprint training (MST) group (n = 8) based on initial sprint performance over 20-meters. Sprint performance was assessed during maximal 20-meter sprint efforts via a radar gun (36 Hz), with velocity-time data used to derive force-velocity characteristics and split times. All subjects then completed a 7-week in-season training intervention consisting of maximal sprinting (MST & CST groups) and assisted sprinting (CST only), along with their usual football specific exercises.

**Results:**

Moderate to large pre-post within group effects (−0.65 ≤ ES ≥ 0.82. *p* ≤ 0.01) in the CST group for relative theoretical maximal force (F_0_) and power (P_max_) were reflected in improved sprint performance from 0–20 m, thereby creating a more force-oriented F-v profile. The MST group displayed statistically significant pre-post differences in sprint performance between 10–20 m only (ES = 0.18, *p* = 0.04). Moderate to high relative reliability was achieved across all sprint variables (ICC = 0.65–0.91), except for the force-velocity slope (S_FV_) and decrement in ratio of forces (D_RF_) which reported poor reliability (ICC = 0.41–0.44), while the CST group exceeded the pre-post minimal detectable change (MDC) in most sprint variables suggesting a ‘true change’ in performance across the intervention.

**Conclusion:**

It is concluded that implementing a short-term, combined sprint training intervention consisting of assisted and maximal sprint training methods may enhance sprint mechanical characteristics and sprint performance to 20-meters in junior AF players.

## Introduction

High-speed running and sprinting are key requirements in Australian football (AF) (Coutts et al., 2010). Within a junior AF setting, sprint characteristics and performance have been shown to differentiate between ability levels including those drafted and non-drafted into the professional ranks of the sport (*i.e.,* Australian Football League; AFL) (Burgess, Naughton & Hopkins, 2012; Edwards et al., 2020b; Pyne et al., 2005). Sprint performance (20-meter) is also measured in the standardized test battery for those athletes who attend the annual AFL draft combine (Pyne et al., 2005), therefore exploring sprint characteristics and specific training methods to improve sprint performance in aspiring junior AF players would be useful for practitioners.

Typically, sprint characteristics in team sport fitness batteries, including the AFL, are described by intermediate split times (*i.e.,* 5-meter and 10-meter time) and overall sprint time, thereby providing a quantitative measure of performance (Pyne et al., 2005). However, this approach to sprint assessment is limiting in nature as it does not explain the underpinning biomechanical and neuromuscular mechanisms contributing to performance. More recently, a macroscopic inverse dynamics approach to sprint assessment known as sprint force-velocity profiling has been utilized in team sport settings to explain and quantify the force, velocity and power characteristics contributing to sprint performance (Edwards et al., 2021a; Morris, Weber & Netto, 2020; Watkins et al., 2021). This approach has helped practitioners better understand the individual force-velocity characteristics of the athlete and the influence mechanical characteristics have on sprint performance. The key mechanical variables obtained from sprint force-velocity profiles include theoretical maximal force (F_0_), theoretical maximal velocity (v_0_) and theoretical maximal power (P_max_) (Samozino et al., 2016), each of which characterize independent neuromuscular characteristics.

Training methods to enhance sprint performance are often focussed on applying progressive overload to a component of the F-v continuum *via* modalities such as resisted sprint training, plyometrics and traditional strength training (Edwards et al., 2022; Haugen et al., 2019b), however no previous research has investigated the effect of using assisted sprint training within a junior AF cohort and its long-term benefit to improving sprint mechanical characteristics. Assisted sprint training is based on overloading the velocity component of the F-v relationship (van den Tillaar, 2021) and is a term often used synonymously with overspeed or supramaximal training, where the aim is to create running velocity greater than what can be achieved in unassisted voluntary conditions (Kristensen, VandenTillaar & Ettema, 2006). Seminal studies in assisted sprint training identified supramaximal velocities (10.36 ± 0.31m/s) was significantly correlated with stride rate (*r* = 0.63, *p* < 0.01), while average net resultant force in the concentric phase correlated with stride length (*r* = 0.65, *p* < 0.01) (Mero & Komi, 1986); this is thought to serve as a specific force indicator in sprinting. It was highlighted by the same authors that electromyography (EMG) increases in lower limb muscles prior to ground contact provided a higher level of muscle stiffness and pre-activation of lower limb muscles was a result of centrally driven recruitment of motor units upon ground contact to withstand supramaximal velocities (Mero & Komi, 1986; Mero & Komi, 1987a; Mero & Komi, 1987b). Collectively, it is suggested that assisted sprint training may provide and additional stimulus for the neuromuscular system during training to achieve higher running velocities when unassisted (Mero & Komi, 1985).

Assisted sprint training methods include running downhill (Ebben, Davies & Clewien, 2008), using a horizontal pulley system (Clark et al., 2009; Kristensen, VandenTillaar & Ettema, 2006), a portable robotic resistance device *e.g.*, 1080 Sprint™ (Lahti et al., 2020), MuscleLab DynaSpeed™ (van den Tillaar, 2021), or elastic pulling cords, which are a cost effective option to enhance sprint speed (Bartolini et al., 2001; Clark et al., 2009; Corn & Knudson, 2003; Makaruk, Stempel & Makaruk, 2019; Mero & Komi, 1985; Upton, 2011). Several research studies have focussed on the acute effects of assisted sprint training using elastic pulling cords with results identifying positive changes to sprint performance (Bartolini et al., 2001; Clark et al., 2009; Corn & Knudson, 2003; Makaruk, Stempel & Makaruk, 2019; Mero & Komi, 1985). However, limited studies exist on the same training methodology within an interventional setting (Makaruk, Stempel & Makaruk, 2019; Upton, 2011) and the influence on sprint mechanical characteristics and performance. In this regard, previous research has (Upton, 2011) reported increased acceleration performance to 15-yards (13.7-meters), specifically in the first 5-yards (4.6 m), when using an assisted training protocol with elastic pulling cords across a 4-week period. Furthermore, using a similar protocol across a 5-week period (Makaruk, Stempel & Makaruk, 2019), significant (*p* < 0.05) interactions have been identified for running velocity, stride frequency, ground contact time and flight time. Despite the implementation of sprint training methods into various football codes (Nicholson et al., 2021), knowledge about the effects of assisted sprint training are limited yet may be a viable form of non-traditional sprint training for AF players.

Therefore, the purpose of this study was to quantify changes to sprint mechanical characteristics in junior AF players by using a combined sprint training (*i.e.,* assisted and maximal sprinting) methodology which focussed on enhancing the velocity component of the F-v continuum. Our primary aim was to determine the influence of a 7-week combined sprint training intervention on sprint F-v characteristics and performance. We hypothesized that (1) a combined sprint training methodology would enhance sprint mechanical characteristics in unassisted sprinting and create a more velocity-oriented profile compared to maximal sprinting only, due to enhanced neural activation (Mero & Komi, 1985), and (2) due to the pulling force assisting athletes to achieve greater velocities (Bartolini et al., 2001), reduction in overall sprint times would be a result of higher velocities achieved from 10–20 m, compared to 0–10 m.

## Materials & Methods

### Study design and participants

A pre-test *versus* post-test experimental design with two groups was selected to investigate the effects of a combined sprint training intervention (7-weeks) in junior AF players. A power analysis was conducted prior to the study (G*Power 3) (Faul et al., 2007) using the following test details: ‘ANOVA: Repeated measures, within-between interaction’, with an effect size of 0.3, alpha of 0.05 and power of 0.8 , which suggested the total sample size of the study should include 24 participants. Twenty-eight junior male AF players from the same specialist sport academy focusing on Australian football, volunteered to participate in this study. Twenty-two (age: 14.4 ± 0.3 years, body mass 58.5 ± 10.0 kg, and height 1.74 ± 0.08 m) met the inclusion criteria of completing 10-12 sessions (2 sessions per week; ≥70%) within 7 weeks, excluding familiarization and pre and post testing. From these participants, six completed 100% of sessions, five completed 91% of sessions, seven completed 83% of sessions and four completed 75% of all sessions. The data from participants who could not complete post-testing was removed from all statistical analysis. Inclusion criteria included: participants involved in AF and aged under 18 years of age. Exclusion criteria maintained that participants needed to be six-months free of musculoskeletal injuries which may prevent them from performing maximal effort sprints. In their pre-testing questionnaire, the adult guardian acknowledged the participant’s experience with sprinting actions and provided written informed consent before beginning the study. The study was conducted in accordance with the Declaration of Helsinki, and the protocol was approved by the Social and Behavioural Research Ethics Committee at Flinders University (Ethics App Number: 8146).

The 7-week training intervention was created with a combined sprint training (CST) group and maximal sprint training (MST) group. The CST group completed maximal assisted sprint efforts and maximal unassisted sprint efforts, while the MST group performed maximal unassisted sprint efforts only. Depending on the structure of the training session, specific sprint-based exercises (maximal and assisted sprint training) were performed on an indoor basketball court or outdoors on a football field. The MST group did not participate in any assisted sprint training protocols. Familiarization of the sprint training assessment and intervention began four weeks prior to testing and included 4–6 × 10–30 m maximal effort unassisted sprint efforts for both groups and assisted sprint efforts for the CST group once per week. This timeline was selected to ensure participants were exposed to the assisted sprinting stimulus in small doses prior to testing and beginning the intervention and to reduce the risk of injury using this training method (Hicks, 2017). During familiarization sessions for assisted sprinting, players practiced sprinting over distances between 10–20 m with the elastic cord at pulling forces progressing from sub-maximal (50–75% stretch on cord; ∼30–75N) to maximal (100% stretch on cord; ∼90N) using a progressive overload approach. Elastic cord tension was measured using a spring balance at various distances (*i.e.,* 10 m, 12.5 m, 15 m) to determine the percentage of maximal pulling force. No changes were observed when measuring pre and post cord tension. Pre-testing coincided with the conclusion of the pre-season period and start of the competitive season for junior AF teams, while post-testing occurred during the middle of the competitive season.

### Testing procedures

#### Force-velocity profile assessment

The sprint force-velocity profile assessment was performed on an indoor basketball court with participants wearing standard athletic clothing and shoes. Prior to the first sprint trial, participants performed a series of six sprint efforts over 10–20 m progressing from sub-maximal to near-maximal. Participants then performed three 20-meter maximal sprint efforts from a standing start (staggered stance; dominant foot forward) and were encouraged to sprint maximally past the 20-meter marker. Between each sprint attempt there was 5-minute passive recovery period to limit fatigue prior to the next sprint effort. Participants were ranked (1-fastest time, 28-slowest time) according to their mean sprint performance (0–20 m) during pre-testing and then pairwise matched to the CST or MST group creating two balanced groups of 14 participants. Unfortunately, injuries and COVID-19 related health concerns impacted six participants who started the intervention study in the MST group, therefore reducing this group number to eight participants. Pre- and post sprint assessments occurred on a single day.

Velocity measurements were recorded continuously during each attempt using a radar gun. Software provided by the radar device manufacturer (STATs software, Stalker ATS I Version 3.0, Applied Concepts, Richardson, TX, USA) was used to collect raw velocity-time data across each sprint trial. The radar device (Model: Stalker ATS I, 36.6 Hz, Applied Concepts, Richardson, TX, USA) was positioned 5 m directly behind the starting position and at a vertical height of 1 m to approximately align with the subject’s centre of mass. Participants bodymass was assessed using dual force plates (35 cm by 35 cm, PASPORT force plate, PS-2141, PASCO Scientific, Roseville, CA, USA), while standing stature was determined using a stadiometer. Individual data files, anthropometric variables and environmental conditions (*i.e.,* barometric pressure, temperature) were then processed and imported into the ‘shorts’ package (Jovanovic, 2021) written in R language (R Development Core Team, 2020). The ‘shorts’ package uses non-linear least squares regression implemented in the ‘nls’ function in R (Bates & Chambers, 1992; Bates & Watts, 1988). Both R and the ‘shorts’ package are open-source software. Any velocity-time data before the onset of movement or past the total sprint distance was filtered from the analysis. Using an inverse dynamics approach of the subject’s center of mass locomotion, the ‘shorts’ package (Jovanovic, 2021) fits an exponential function to the raw velocity-time data from the radar gun to establish all variables. The biomechanical model and equations of this approach have previously been reported (Samozino et al., 2016) and validated (Morin et al., 2019) when compared with direct measurement of ground reaction forces (GRF) from in-ground force plates and has been used in previous interventional studies (Lahti et al., 2020). Sprint position-time data (*i.e.,* split times) were derived from velocity-time data from the radar and were analyzed separately for each participant to establish sprint force-velocity profiles, and associated sprint mechanical characteristics by following previously validated methods (Samozino et al., 2016). The mean of three maximal sprint trials from each participant was used for statistical analysis.

### Intervention protocol

**Figure 1:**
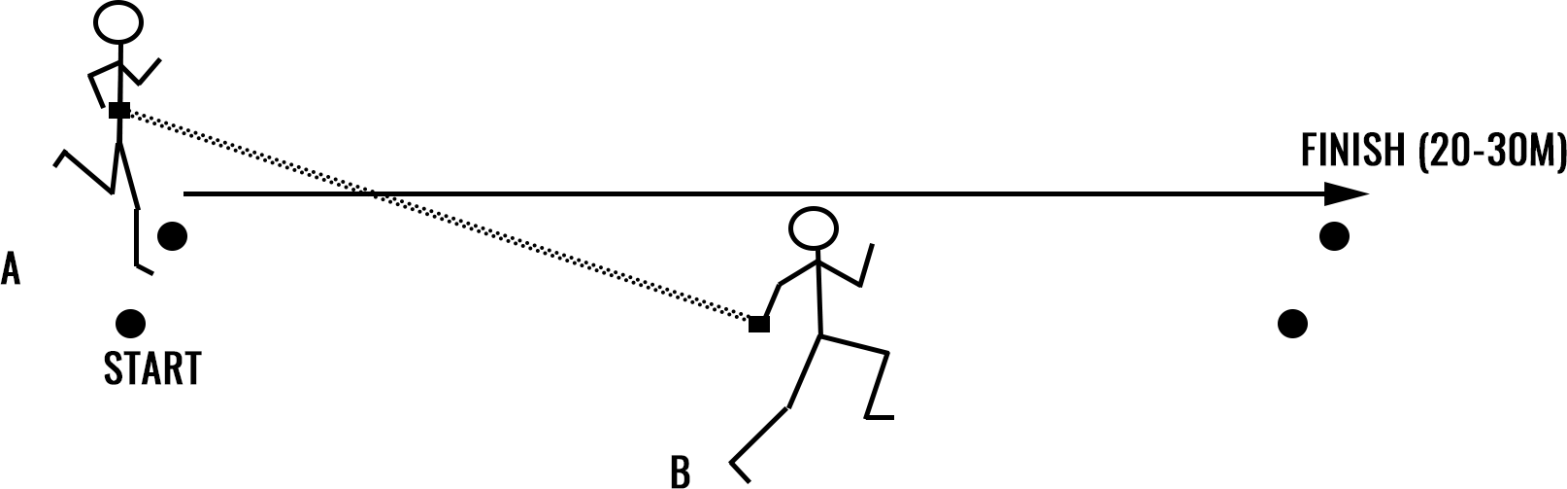
Visual description of assisted sprinting design. (A) subject pulled by elastic cord (attached around the waist); (B) subject pulling the elastic cord (cord held outstretched in hand).

Assisted sprinting was performed using a 6-meter elastic cord (HART Catapult Trainer) harnessed to the waist of the runner. The elastic cord was fully stretched prior to a sprint, thereby establishing a pulling force of approximately 97.5N (± 15N) at 15-meters, as measured by a spring balance, and held in position by the accredited strength and conditioning coach (Australian Strength & Conditioning Association Level 2, ASCA). Cords could not be stretched greater than 15-meters. Upon receiving a 3-second countdown, the harnessed athlete would sprint maximally up to a distance of approximately 20–30 m. Once the harnessed athlete began to sprint, the coach holding the other end of the elastic cord must also run for approximately 10–15 m in the same direction to maintain the highest level of tension on the cord to assist the runner until they reach the required distance (Fig. 1). The coach ran a slight angle (5−10°) to the athlete to ensure the athlete was not impeded by running over the elastic cord. Despite the coaches best efforts, it is acknowledged the tension on the cord is reduced once the athlete begins to accelerate (Bartolini et al., 2001). After sprinting using the elastic cords, several players provided feedback to the coaches including ‘*I felt like I was catapulted off the start line’* and ‘*sprinting is so easy with the cords.’*

Sessions for all participants (*i.e.,* CST and MST) were conducted and supervised by ASCA coaches and completed twice weekly prior to on-field technical and tactical football session. Prior to all intervention-based training sessions, participants performed a 10-minute warm-up consisting of linear and multi-directional movement patterns, dynamic stretches, mobility, and activation exercises, and progressed from general to more sprint specific exercises (*i.e.,* marching, A-skip, scissor bound). There was no added sprint specific training included in the on-field sessions and overall training volume remained stable across the intervention, thereby maintaining a level of consistency across the study. At the conclusion of each sprint effort, each participant undertook a rest period of approximately 3–5 min to limit fatigue prior to the next sprint. All protocols specific to the 7-week training intervention are outlined in Table 1. Sprint volume between both groups were matched across the duration of the intervention.

**Table 1:** Description of entire training intervention.

Week	Group		Volume/ session	Weekly volume
	CST (Combined sprint training: assisted and maximal sprint training)	MST (Maximal sprint training)		
	Session	Session		
−4 Familiarization	Sub-maximal to maximal unassisted and assisted sprint efforts over 10–30-metres.		100 m	200 m
−3 Pre-Testing	3 × 20-metre sprint efforts. Velocity-time data collected via radar device.		60 m	120 m
−2 & −1	Technical and tactical football sessions only		–	–
1	2 × 20 m AST/1 × 20 m MST	3 × 20 m MST	60 m	120 m
2	3 × 20 m AST	3 × 20 m MST	60 m	120 m
3	3 × 25 m AST	3 × 25 m MST	75 m	150 m
4	3 × 25 m AST/1 × 15 m MST	1 × 15 m, 3 × 25 m MST	90 m	180 m
5	3 × 30 m AST	3 × 30 m MST	90 m	180 m
6	3 × 30 m AST/1 × 30 m MST	4 × 30 m MST	120 m	240 m
7	4 × 30 m AST	4 × 30 m MST	120 m	120 m
8 Post-Testing	3 × 20-metre sprint efforts. Velocity-time data collected via radar device.		60 m	60 m

**Notes.**

AST, Assisted sprint training.

### Statistical analysis

Statistical analyses were performed in R (v3.6.1; R Foundation for Statistical Computing, R Core Team, Vienna, Austria), in the RStudio environment (v1.2.519; RStudio, Inc., Boston, MA, USA) using various statistical packages. All descriptive data are presented as mean ± standard deviation (SD) and were assessed for normality and variance using the Shapiro–Wilks and Levene’s test respectively. Independent samples *t*-tests were used to determine between group differences at pre-test for sprint F-v characteristics and split-times. Intraclass correlation coefficient (ICC) with 95% confidence limits were used to assess relative reliability of force-velocity and split times for sprint trials (Hopkins, 2000). To account for typical fluctuations in sprint performance between testing sessions, the minimal detectable change (MDC) at 90% confidence intervals, was used to determine the minimum level of change necessary to represent a ‘true’ performance change, rather than random measurement error and was calculated as 1.645 × standard error of measurement (SEM) × $\sqrt{2}$ (Furlan & Sterr, 2018; Haley & Fragala-Pinkham, 2006). The MDC% was defined as (MDC/ =*x*) × 100 (Flansbjer et al., 2005). Thresholds for evaluation of intraclass correlation coefficients were quantified using the following scale: 0.20−0.49 *poor,* 0.50−0.74 *moderate*, 0.75−0.89 *high*, 0.90−0.98 *very high* and ≥ 0.99 *extremely high* (Hopkins et al., 2009). To assess the effect of assisted sprint training a 2 (pre-post-test: repeated measurements) × 2 (group: CST, MST) ANOVA was performed. Standardized effect sizes (Cohen’s *d*) were assessed pre-post training for all sprint force-velocity variables and split-times were determined using a pooled standard deviation approach from both groups with 95% confidence limits. Magnitudes of effect size changes were interpreted using the following values: trivial (<0.20), small (0.20 ≤ 0.60), moderate (0.60 ≤ 1.20), large (1.20 ≤ 2.00) and extremely large (*>* 2.00) (Cohen, 1988). In addition, a one-way ANOVA with repeated measures per group was conducted to identify changes per group. An alpha value of *p* ≤ 0.05 was used to indicate statistical significance.

## Results

**Figure 2:**
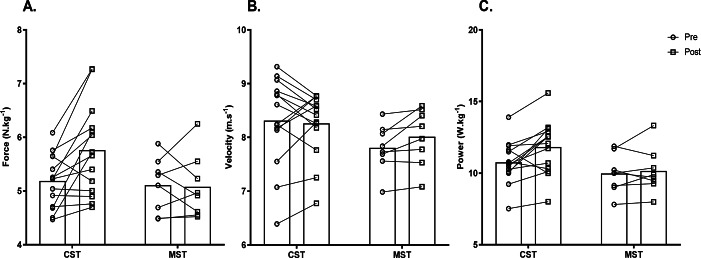
Pre-post changes to sprint force-velocity characteristics and split times after the 7-week training intervention. (A) Relative theoretical maximal force; (B) Theoretical maximal velocity; (C) Relative theoretical maximal power. (CST, combined sprint training group, MST, maximal sprint training group).

**Figure 3:**
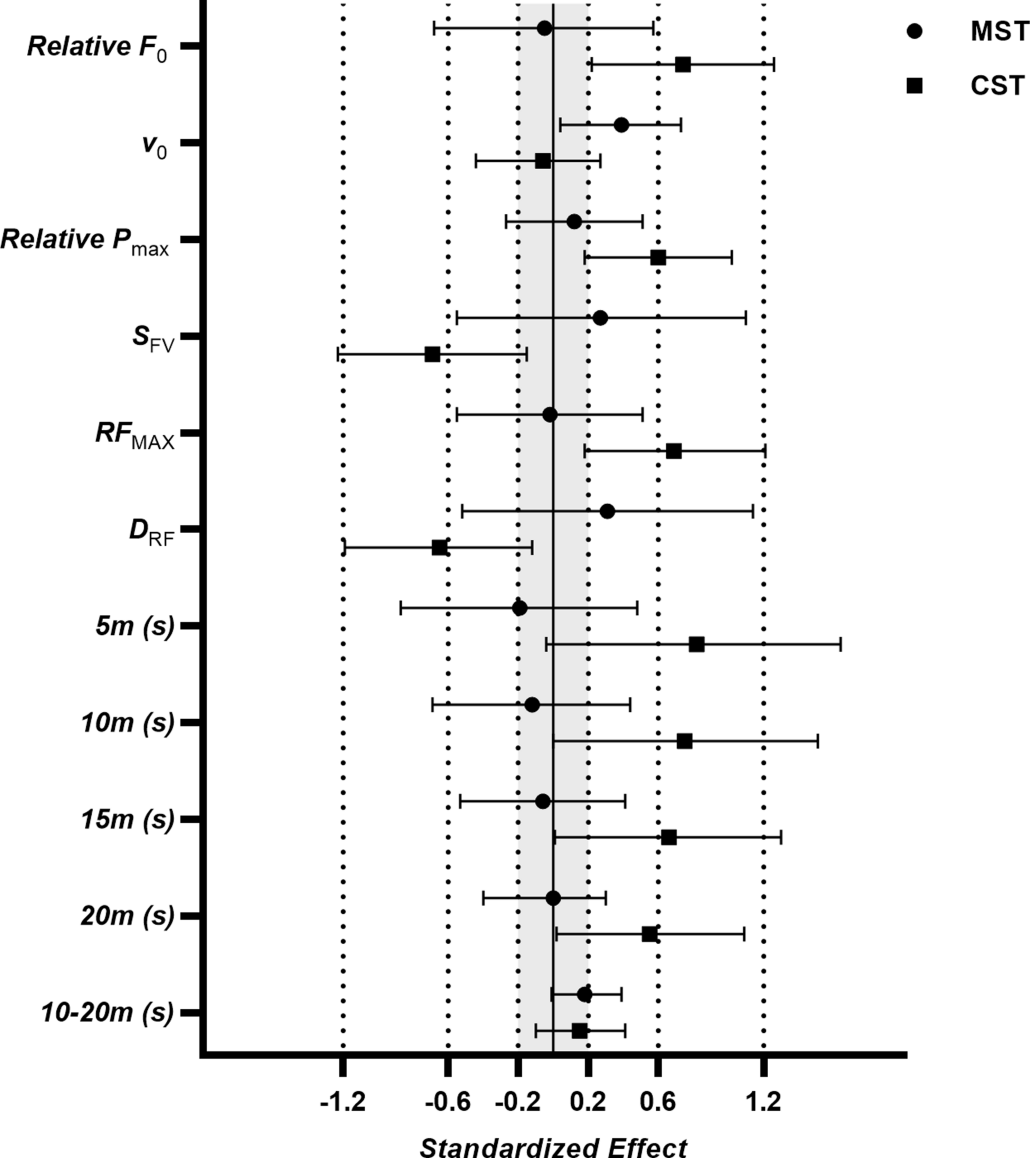
Within-group pre-post effect sizes for sprint force-velocity characteristics and split times across the 7-week intervention. (*F*_0_, theoretical maximal force; v_0_, theoretical maximal velocity; P_max_, theoretical maximal power; S_*FV*_, force-velocity slope; RF_*MAX*_, maximum ratio of forces; D_*RF*_, decrement in ratio of forces; CST, combined sprint training group; MST, Maximal sprint training group).

**Figure 4:**
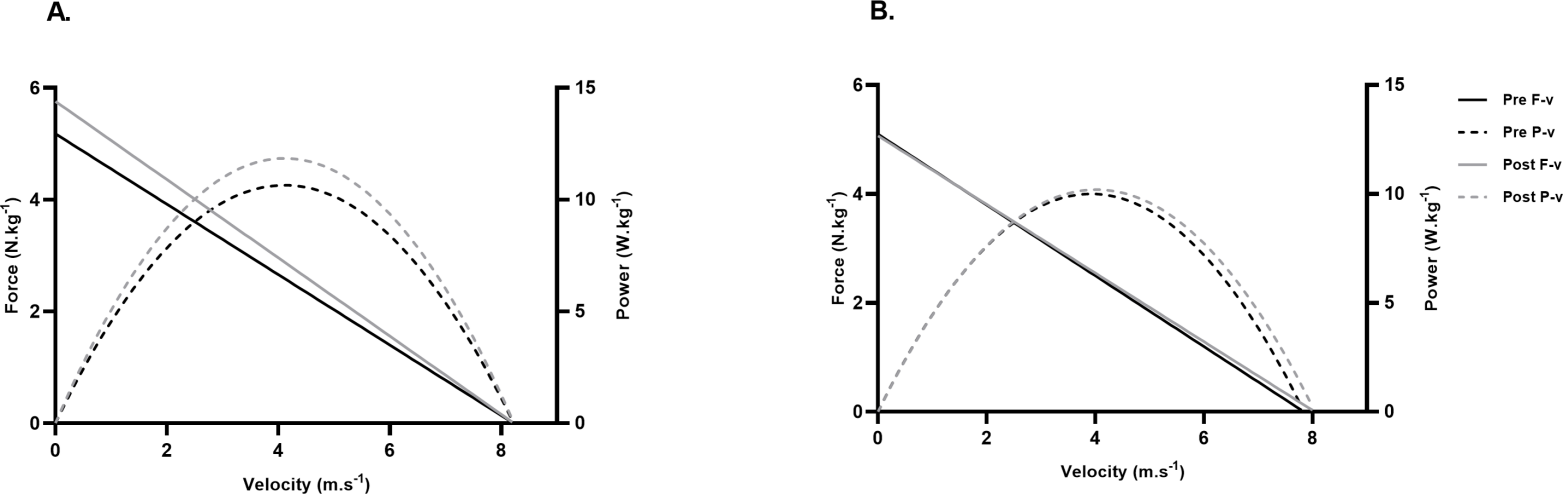
Mean pre-post sprint force-velocity-power profile of 20-meter sprint performance. (A) Combined sprint training (CST) group; (B) maximal sprint training (MST) group. (F-v, force-velocity; P-v, power-velocity).

Shapiro–Wilks and Levene’s test confirmed normality and homogeneity of variance for all F-v variables. All results are reported in Tables 2–3 and Figs. 2–4. The mean session completion rate in the CST and the MST group were 78.6% and 85.7% respectively. At the pre-test, no significant differences were observed between groups (MST *vs* CST) for all sprint force-velocity (*t* ≤ 1.87, *p* ≥ 0.07) or split-times variables (*t* ≤ 1.59, *p* ≥ 0.12).

**Table 2:** Reliability measures and minimal detectable change for sprint force-velocity variables and split-times.

Variable	Relative F_0_ (N kg^−1^)	v_0_ (m s^−1^)	Relative P_*max*_ (W kg^−1^)	Relative S_*FV*_ (N s m^−1^ kg^−1^)	RF_MAX_ (%)	D_**RF**_ (% m s^−1^)	5 m (s)	10 m (s)	15 m (s)	20 m (s)	10–20 m (s)
ICC	0.65 (0.29, 0.83)	0.72 (0.41, 0.88)	0.85 (0.62, 0.96)	0.44 (0.20, 0.74)	0.71 (0.41, 0.90)	0.41 (0.16, 0.72)	0.65 (0.47, 0.76)	0.79 (0.65, 0.88	0.85 (0.75, 0.91	0.89 (0.80, 0.95)	0.91 (0.83, 0.96)
SEM	0.10	0.15	0.30	0.01	0.005	0.001	0.01	0.02	0.03	0.04	0.02
MDC	0.24	0.36	0.72	0.04	0.01	0.003	0.05	0.08	0.10	0.13	0.05
MDC%	4.71	4.54	6.88	6.30	3.36	6.27	2.65	2.59	2.60	2.65	3.17

**Notes.**

ICC intraclass correlation coefficient (95% confidence intervals) SEM standard error of measurement MDC minimal detectable change F_0_ theoretical maximal force v_0_ theoretical maximal velocity P_max_ theoretical maximal power S_FV_ force-velocity slope RF_MAX_ maximum ratio of forces D_RF_ decrement in ratio of forces

**Table 3:** Pre-post sprint force-velocity variables and split times for within and between-group comparisons.

Variable	Group	PRE Mean ± SD	POST Mean ± SD	Within-group ES (pre-post) *P* value	% Δ ± SD	Between-group-time ES ± 95% CL, *F* value, *P* value
Relative F_**0**_ (N.kg^−1^)	CST MST	5.18 ± 0.49 5.10 ± 0.51	5.76 ± 0.84 5.07 ± 0.59	0.74 (0.22, 1.26), 0.005** −0.05 (−0.68, 0.57), 0.85	11.19 ± 12.52 −0.40 ± 8.47	Group ES: −0.11 (−0.95,0.73), *F* = 0.07, *p* = 0.78 Time ES: 0.84 (0.12,1.55), *F* = 5.57, *p* = 0.02* Int ES: −0.88 (−2.06,0.31), *F* = 2.24, *p* = 0.14
v_**0**_ (m.s^−1^)	CST MST	8.31 ± 0.83 7.80 ± 0.43	8.25 ± 0.60 8.01 ± 0.52	−0.06 (−0.41, 0.27), 0.69 0.39 (0.04, 0.73), 0.03*	−0.25 ± 6.08 2.60 ± 2.87	Group ES: −0.77 (−1.66,0.12), *F* = 3.07, *p* = 0.08 Time ES: −0.08 (−0.84,0.67), *F* = 0.04, *p* = 0.82 Int ES: 0.39 (−0.86,1.65) *F* = 0.40, *p* = 0.53
Relative P_**max**_ (W.kg^−1^)	CST MST	10.75 ± 1.47 9.96 ± 1.36	11.80 ± 1.86 10.15 ± 1.57	0.60 (0.18, 1.02), 0.007** 0.12 (−0.27, 0.51), 0.52	10.04 ± 11.56 1.94 ± 7.28	Group ES: −0.46 (−1.30,0.38), *F* = 1.22, *p* = 0.27 Time ES: 0.61 (−0.10,1.33), *F* = 2.99, *p* = 0.09 Int ES: −0.50 (−1.69,0.68), *F* = 0.73, *p* = 0.39
Relative S_**FV**_ (N.s.m^−1^.kg^−1^)	CST MST	−0.63 ± 0.09 −0.65 ± 0.05	−0.70 ± 0.12 −0.63 ± 0.07	−0.64 (−1.23, 0.15), 0.01* 0.27 (−0.55, 1.10), 0.48	12.96 ± 16.33 −2.46 ± 10.24	Group ES: −0.26 (−1.13,0.62) *F* = 0.35, *p* = 0.55 Time ES: −0.79 (−1.53, −0.04), *F* = 4.57, *p* = 0.03* Int ES: −0.50 (−1.69,0.68), *F* = 2.53, *p* = 0.11
RF_**MAX**_	CST MST	0.39 ± 0.02 0.39 ± 0.02	0.42 ± 0.03 0.39 ± 0.03	0.69 (0.18, 1.21), 0.008* −0.02 (−0.55, 0.51), 0.91	6.06 ± 7.31 −0.14 ± 5.21	Group ES: −0.23 (−1.08, 0.62), *F* = 0.30, *p* = 0.58 Time ES: 0.71 (−0.01, 1.43), *F* = 3.96, *p* = 0.05* Int ES: −0.74 (−1.94, 0.46), *F* = 1.54, *p* = 0.22
D_**RF**_	CST MST	−0.05 ± 0.01 −0.06 ± 0.01	−0.06 ± 0.01 −0.06 ± 0.01	−0.65 (−1.19, −0.12), 0.01* 0.31 (−0.52, 1.14), 0.44	11.91 ± 15.48 −2.63 ± 9.73	Group ES: −0.30 (−1.18, 0.62), *F* = 0.47, *p* = 0.49 Time ES: −0.75 (−1.50, 0.00), *F* = 4.09, *p* = 0.04* Int ES: 0.96 (−0.29, 2.20), *F* = 2.41, *p* = 0.12
Split time 0–5 m (s)	CST MST	1.72 ± 0.09 1.74 ± 0.09	1.64 ± 0.09 1.75 ± 0.09	0.82 (−0.04, 1.64), 0.02* −0.19 (−0.87, 0.48), 0.55	−4.39 ± 7.31 1.09 ± 4.39	Group ES: 0.11 (−0.72, 0.95), *F* = 0.07, *p* = 0.78 Time ES: −0.80 (−1.51, −0.09), *F* = 5.16, *p* = 0.02* Int ES: 0.97 (−0.21,2.15), *F* = 2.75, *p* = 0.10
Split time 0–10 m (s)	CST MST	2.60 ± 0.14 2.64 ± 0.13	2.50 ± 0.13 2.65 ± 0.11	0.75 (0.00, 1.51), 0.02* −0.12 (−0.69, 0.44), 0.65	−3.76 ± 6.15 0.69 ± 3.60	Group ES: 0.24 (−0.60, 1.07), *F* = 0.33, *p* = 0.56 Time ES: −0.73 (−1.44, −0.01), *F* = 4.23, *p* = 0.04* Int ES: 0.84 (−0.35,2.02), *F* = 2.04, *p* = 0.16
Split time 0–15 m (s)	CST MST	3.36 ± 0.18 3.42 ± 0.17	3.24 ± 0.16 3.43 ± 0.13	0.66 (0.01, 1.30), 0.02* −0.06 (−0.53, 0.41), 0.79	−3.24 ± 5.25 0.37 ± 2.99	Group ES: 0.34 (−050, 1.18), *F* = 0.67, *p* = 0.41 Time ES: −0.64 (−1.36, 0.08), *F* = 3.25, *p* = 0.07 Int ES: 0.70 (−0.49,1.89), *F* = 1.39, *p* = 0.24
Split time 0–20 m (s)	CST MST	4.05 ± 0.22 4.15 ± 0.20	3.94 ± 0.19 4.15 ± 0.17	0.55 (0.02, 1.09), 0.02* 0.00 (−0.40, 0.38), 0.96	−2.78 ± 4.54 0.10 ± 2.55	Group ES: 0.43 (−0.42, 1.28), *F* = 1.04, *p* = 0.31 Time ES: −0.55 (−1.27, 0.17), *F* = 2.37, *p* = 0.13 Int ES: 0.56 (−0.64, 1.76), *F* = 0.88, *p* = 0.25
Split time 10–20 m (s)	CST MST	1.45 ± 0.09 1.51 ± 0.07	1.43 ± 0.07 1.48 ± 0.07	0.15 (−0.10, 0.41), 0.23 0.18 (−0.01, 0.39), 0.04*	−1.05 ± 3.09 −0.96 ± 1.36	Group ES: 0.65 (−0.22, 1.53), *F* = 2.27, *p* = 0.03* Time ES: −0.17 (−0.92, 0.58), *F* = 0.21, *p* = 0.57 Int ES: 0.01 (−1.23,1.25), *F* = 0.00, *p* = 0.98

**Notes.**

ES effect size CL confidence limits CST combined sprint training group MST Maximal sprint training group F_0_ theoretical maximal force v_0_ theoretical maximal velocity P_max_ theoretical maximal power S_FV_ force-velocity slope RF_MAX_ maximum ratio of forces D_RF_ decrement in ratio of forces

*p ≤ 0.05, **p ≤ 0.01.

Reliability measures, standard error of measurement (SEM) and minimal detectable change (MDC) data for sprint mechanical characteristics and split times are presented in Table 2. The intraclass correlation coefficients (ICC) for sprint mechanical characteristics and split times ranged from moderate to high (ICC = 0.65−0.91), except for the force-velocity slope (S_FV_) and decrement in ratio of forces (D_RF_) which both reported poor reliability measures (ICC = 0.41−0.44). The minimal detectable change (%) across sprint mechanical variables and split times ranged from 2.59−6.88%. The CST group exceeded the MDC for most variables suggesting a ‘true change’ in performance across the intervention except for v_0_, split time to 15 m, 20 m and 10–20 m. Changes in the MST group did not exceed those of the MDC.

Significant time*group interaction effect was found for relative F_0_, relative S_FV_, RF_MAX_, D_RF_ and split time to 5-meters and 10-meters (*F* ≥ 3.96, *p* ≤ 0.05, −0.80 ≤ ES ≥ 0.84) (Table 3, Figs. 2–3). Changes to absolute values of mechanical characteristics can be found in supplemental files (Table S1), highlighting no significant group effects were found between variables (*F* ≤ 3.07, *p* ≥ 0.08) (Table 3). *Post hoc* comparison revealed the MST group significantly increased v_0_ and split time from 10–20 m only (0.18 ≤ ES ≥ 0.39, *p* ≤ 0.04), while significant increases in sprint mechanical characteristics and sprint performance were reported in the CST group for almost all variables (−0.64 ≤ ES ≥ 0.82, *p* ≤ 0.01) (Table 3, Figs. 2–3). Analysis of sprint performance in the CST group showed significant pre-post % changes at all sprint distances to 20-meters (2.78−4.49%)(Table 3, Fig. 2). No significant differences (*p* > 0.05, ES = −0.10) were noted for body mass (ES =−0.10, 95%CI [−0.12, −0.06], *p* = 0.06) between testing days. Mean group pre-post changes between sprint force-velocity profiles over 20-meters are presented in Fig. 4. Pre-post analysis of the F-v profile identified 12/14 participants (85%) in the CST group had a more force-oriented profile post-intervention, compared with 3/8 of participants (38%) in the MST group.

## Discussion

The purpose of this study was to investigate the effects of a 7-week combined sprint training intervention (assisted sprint and maximal sprint training) on sprint mechanical characteristics and performance in junior AF players. To the best of our knowledge this is the only study which has reported the effects of this type of training intervention in conjunction with a focus on the mechanical characteristics of the sprint F-v profile. The main findings of this study identified a combined sprint training approach significantly improved sprint performance (*i.e.,* reduced sprint time over 20-meters), whereas minor changes were observed for mechanical and performance characteristics in the maximal sprint training group. Reduced sprint times across all distances (2.78−4.49%) in the CST group were reflected in significant changes to relative theoretical maximal force (10.04%) and power (11.19%), which were greater than the minimal detectable change for each variable. Maximal sprint training only elicited significant changes to v_0_ (2.60%) and split time from 10–20m (0.96%) in the MST group, highlighting the effectiveness and utility of this training method to improve maximal velocity in field-based sports. The results from this intervention study suggests a combined sprint training approach may be a viable option for junior AF players when attempting to improve sprint performance during the in-season period.

Although sprint performance is not the sole predictor of success in Australian football (*i.e.,* tactical & technical abilities, physiological qualities), developing this quality appears conducive for progressing to higher levels of the sport suggesting understanding and then developing sprint mechanical characteristics is important for sports performance coaches (Burgess, Naughton & Hopkins, 2012; Edwards et al., 2020b; Robertson, Woods & G, 2015). In reference to our first hypothesis, we identified that a combined sprint training approach created a more force-dominant F-v profile, leading to greater acceleration ability due to pre-post changes to relative theoretical maximal force (ES: 0.74) and power (ES: 0.60) (Fig. 4). This contradicted our initial hypothesis as it appears significant changes to mechanical and performance characteristics in the initial steps of the sprint (*i.e.,* 0–10 m) is due to the transfer of training effect of the supramaximal velocity stimulus from the elastic cord in the early acceleration phase. This biomechanical change in performance is supported in the results by the moderate effect sizes for relative maximal force and split time from 0–5 m (−0.80 ≤ ES ≥ 0.84). Furthermore, motor learning research details greater transfer or ‘crossover’ to normal sprinting occurs when the biomechanics target specific technical sprint elements (Haugen et al., 2019b), in this case a greater exposure to supramaximal velocities at the start of the sprint effort. As previously reported (Bartolini et al., 2001), the pulling force of the elastic cord most likely lost tension relative to the athlete’s bodyweight at distances greater than 15-meters. suggesting the stimulus was likely negligible when in an upright position, *i.e.,* approximately 10–20 m. It can therefore be inferred that the mechanical changes affecting early acceleration has led to faster split times across the sprint effort except for the 10–20 m flying segment. These findings are important considering previous studies in Australian football have reported high numbers of acceleration-based efforts in elite male players identifying the importance of developing mechanical characteristics (Varley, Gabbett & Aughey, 2013).

Previous intervention studies involving male AF players of similar ages as those in this study (Cahill et al., 2020; Edwards et al., 2022), have reported resisted sprint training using sleds had significant effects on relative theoretical maximal force values (ES: 0.63−1.19) with the greatest performance change occurring in the first 10m of the sprint. It was also suggested to improve sprint performance, junior AF players should develop a force-oriented mechanical profile (Edwards et al., 2020a); which occurred in the CST group during this study, despite using velocity as a speed specific stimulus (Kristensen, VandenTillaar & Ettema, 2006). This is a new finding and suggests a CST approach to sprint performance may provide a similar neuromuscular adaptation to resisted sprint training in adolescent AF populations. Furthermore, large changes to P_max_ in the CST group suggests over this sprint distance, the improvements in F_0_ may be of greater importance compared to v_0_ when trying to improve P_max_ and sprint performance. This may therefore inform practitioners which side of the F-v continuum to place a greater focus on when attempting to improve sprint performance in junior AF players.

Force-velocity profiles and their associated variables have not previously been reported in assisted sprint training interventions using elastic pulling cords, however the sprint performance changes in this study as measured *via* split times align with previous findings (Makaruk, Stempel & Makaruk, 2019; Upton, 2011). Other studies have reported significant effects to early acceleration (<15 m) performance with female college sport athletes using this approach, yet with no reference to the F-v profile, along with an increased mean centre of mass velocity (6.37% Δ), increases in stride frequency (Hz) (5.48% Δ), and decreases in contact time (ms) (8.39% Δ) following a 5-week assisted sprint training programme. Across studies, elastic pulling cords increased mean velocity to 5-yards (10.07% Δ), yet relatively small velocity changes to 25-yards (2.07% Δ) (Upton, 2011). These changes were thought to be the result of enhance neuromuscular response in the early steps of acceleration across the 4-week (12-session) intervention. The difference in our findings compared to previous studies (Makaruk, Stempel & Makaruk, 2019; Upton, 2011) may also be due to a measure of mean velocity across the sprint effort, differences in pulling force, the experience level of the participants (*i.e.,* junior AF players compared to college level athletes) or training volume and intensities used within the intervention.

Our second hypothesis was not confirmed as sprint performance in the CST group did not achieve statistical significance for the split time from 10–20 m. Changes in sprint performance between 10–20 m were evident in the MST group only. These results identify how pulling force from the elastic cords has likely influenced the rate of acceleration at the instant the athlete overcomes inertia yet provided limited assistance to improve velocity adaptations in this segment of the sprint. This was not the case in the MST group where significant changes to v_0_ and split time from 10–20 m were identified, suggesting greater volume and exposure to maximal sprint training performed by these players established greater neuromuscular adaptations impacting this aspect of sprint performance (Haugen et al., 2019b), along with velocity specific adaptations, such as greater vertically directed support forces which have been shown to enhance maximal velocity (Kugler & Janshen, 2010; Weyand et al., 2000). While not the focus of this study, this finding is a consideration for speed development in AF due to the demand for high-speed running (>5.5 m s^−1^) across the duration of the game (70–110 m min^−1^) which has been reported to differentiate between ability levels (Johnston et al., 2018). Although our pre-testing data did not show significant between-group differences for v_0_ (*p* = 0.07), the lower initial values for this variable in the MST group may also suggest participants may have had a velocity-deficit when compared with the CST group and by engaging in maximal sprint training, reduced this mechanical imbalance across the 7-week intervention.

It should be noted that improved sprint performance along with increased relative maximal power in the CST group may have established a more *optimal* F-v profile for this cohort of junior AF players (Samozino et al., 2021). The individual optimal sprint F-v profiles depends largely on P_max_ and to a lesser degree on sprint distance and the interindividual variability in F-v characteristics. Recent research (Samozino et al., 2021) identified as sprint distance was reduced (<15-meters) the optimal F-v profile would become oriented towards force capabilities (*i.e.,* force dominant), whereas, as sprint distance increased (>15-meters) velocity capabilities would be of greater importance to sprint performance and the optimal profile would orient towards being velocity dominant. This is largely supported in our findings when considering pulling force in the CST group appears to be maximized in the initial stages of the sprint effort, however, may also identify this particular group of adolescent AF players exhibit a force-deficit in a sprint context. From a practical perspective, this identifies a potential *window of trainability* to improve maximal power by targeting the force side of the F-v continuum using a combined sprint training approach to *optimize* the mechanical sprint F-v profile.

Investigating the associated sprint mechanical characteristics influencing performance was also important to consider in this study. Significant within-group effects and pre-post changes in the CST group to the maximum ratio of forces (RF_MAX_) suggests changes to force application during sprint performance may have occurred across the training intervention. Previous research (Morin, Edouard & Samozino, 2011) suggests the increase in RF_MAX_ would result in a more horizontally directed ground reaction force in the initial steps of the acceleration thereby directly affecting acceleration capabilities according to Newton’s laws of motion. Furthermore, Morin, Edouard & Samozino (2011) reported an increase in ratio of force (%) is a result of improving the angle and technical ability at which antero-posterior force compared to the corresponding total ground reaction force (*F*_*TOT*_) is averaged over the support phase. Therefore, for the same magnitude of force applied to the ground, the horizontal change in velocity during the stance phase will improve due the orientation of the ground reaction force vector (Bezodis et al., 2021) which may have led to a reduction in all split times in the CST group. Significant changes to decrement of ratio of forces (D_RF_) and relative F-v slope (S_FV_) were reported in the CST group. Changes to D_RF_ highlight how the natural decrease in ratio of forces as running velocity increases has likely been altered due to the assistive pulling force from the elastic cord, whereas the S_FV_ describes the athlete’s individual ratio of force (*i.e.,* acceleration) in reference to velocity (*i.e.,* maximal speed). However, due to the absolute reliability confidence intervals of these variables, we cannot make conclusive statements concerning the utility for the D_RF_ (ICC =0.41) and S_FV_ (ICC = 0.44) to inform practice within this intervention study only.

This experimental study has several strengths. Sprint mechanical characteristics on junior Australian football players can provide valuable insights into the physical capabilities of these athletes, specifically in regard to their neuromuscular output. Such a study can help the sport and strength & conditioning coach design more effective training programs, as well as identify areas where individual players may need to focus on improvement across the force-velocity continuum. Additionally, the results of the study may identify how mechanical profiling can be used to track and monitor changes in the players’ biomechanical and technical sprint abilities across the competitive season. This study has also identified alternate sprint-specific training methods to enhance performance within a football context. Finally, there are a limited number of studies exploring sprint mechanical profiling in youth populations and therefore this adds original knowledge to the growing literature.

There are also limitations in this study which should be acknowledged. Across the duration of the intervention there was limited monitoring of velocity changes in assisted sprint conditions in the CST group. Although the elastic cord tension was measured during the intervention, individual velocity data was not measured for each participant which would have provided greater information about the percentage above maximal velocity each player achieved during the training sessions, thereby potentially highlighting the variability of the training method. Furthermore, despite previously identifying the non-constant pulling force on the athlete while using elastic cords to achieve a supramaximal stimulus, without having a budget to purchase several portable robotic devices with constant pulling force, *i.e.,* 1080 Sprint™, elastic cords may still be a viable option for AF coaches. Also, a power analysis was conducted prior to the study and the desired number of subjects was initially met (*n* = 28), however due to injuries and COVID-19 health implication several participants could not complete the intervention (*n* = 22) and the study became underpowered which may undermine some of the results. *Post-hoc* analysis using 22 subjects therefore provides a power level of only 0.76, which highlights differences between the means will only be detected 76% of the time. Future studies using a larger sample size would therefore provide greater certainty of results. We were also concerned with the poor reliability (ICC = ≤ 0.44) regarding S_FV_ and D_RF_, which is in line with previous research (Haugen, McGhie & Ettema, 2019a). The D_RF_ is the combination of maximum velocity and relative acceleration, and therefore has an interdependence on the individual slope of the force-velocity (S_FV_) relationship. Typically, as one value moves up (*i.e.,* relative force), the other value will likely move down (*i.e.,* velocity) changing the S_FV_ value. Therefore, slight changes in initial acceleration of the sprint effort, *i.e.,* 0–5 m, will reduce the reliability of the velocity-time data from the radar gun (or laser gun), which has previously been identified as a methodology concern (Bezodis, Salo & Trewartha, 2012). Furthermore, small changes in velocity-time data between trials will likely be amplified in derived F-v values, which again places an importance on participant familiarisation of the testing protocol. Also, the adolescent aged population group involved in this study may limit the transfer of findings to senior level AF players. Although maturation is highly individual, studies have shown changes to sprint performance can be influenced by an individual’s chronological age relative to their age at peak height velocity (PHV) and maturation offset (Colyer et al., 2020; Edwards et al., 2021a; Edwards et al., 2021b; Malina et al., 2020; Mirwald et al., 2002). A final limitation was that we did not directly measure pre-post stride kinematics (step-length/step frequency) or muscle activity (EMG) of the lower limbs as has occurred in previous assisted sprint training studies (Mero & Komi, 1986; Mero & Komi, 1987b; van den Tillaar, 2021). This information would have provided a greater understanding of how variables such as stride length, stride frequency, contact time, flight time, joint-segment changes and motor unit recruitment were influenced by mechanical changes due to assisted and maximal sprint training. Combining the mechanical data from F-v profiling, use of a portable robotic device with constant pulling force, plus obtaining stride kinematics and EMG data, would provide greater insight into adaptations caused across the intervention and is worthy of future research.

## Conclusions

Developing sprint ability in junior Australian football players appears to be advantageous for on-field performance and potential selection in the annual Australian Football League national draft, therefore understanding the most effective training methods to improve this quality is important for practitioners. Based upon the findings of the present study, we conclude that a 7-week combined sprint training intervention using assisted (elastic pulling cord) and maximal sprint training methods, may be a more appropriate methodology to enhance various sprint mechanical characteristics and improve sprint performance over 20-meters compared to a traditional maximal sprint training approach. Upon completing familiarization, a progressive overload approach of combined sprint training lasting approximately 15–20 min, starting at 40-meters (total volume) of assisted sprinting and progressing to 120-meters (total volume) of assisted sprinting, could be implemented in the warm-up period prior to football-specific exercises. Practitioners are encouraged to use assisted and maximal sprint training methods in a combined training protocol to create a more force-oriented F-v profile due to significant changes to relative theoretical maximal force and power in junior Australian football players. Coaches should however be cautious when implementing this training modality and ensure familiarisation has been performed by all players to reduce the risk of injury.

##  Supplemental Information

10.7717/peerj.14873/supp-1 Supplemental Information 1 Absolute and relative pre-post sprint force-velocity variables and split times for within and between-group comparisons

10.7717/peerj.14873/supp-2 Supplemental Information 2 Raw data

## Additional Information and Declarations

### Data Availability

The following information was supplied regarding data availability: The raw data is available at OSF: Hicks, Dylan. 2022. “The Effect of a Combined Sprint Training Intervention on Sprint Force-Velocity Characteristics in Junior Australian Football Players.” OSF. November 2. doi: 10.17605/OSF.IO/NVKQ2.
