# Supplementary material for: The effect of a combined sprint training intervention on sprint force-velocity characteristics in junior Australian football players
Source: PeerJ. 2023 Mar 15;11:e14873. doi: 10.7717/peerj.14873 (PMC10024483; doi:10.7717/peerj.14873)
Supplement: Supplemental Information 1 [file peerj-11-14873-s001.docx]

**Table 1. Absolute and relative** **pre-post sprint force-velocity variables and split times for within and between-group comparisons.**

| **Variable** | **Group** | **PRE**  **Mean ± SD** | **POST**  **Mean ± SD** | **Within-group ES (pre-post)**  **P value** | **%Δ ± SD** | **Between-group-time ES ± 95% CL, F value, P value** |
| --- | --- | --- | --- | --- | --- | --- |
| **Absolute F_0_ (N)** | CST  MST | 304.03 ± 49.92  284.70 ± 54.39 | 340.20 ± 76.32  282.42 ± 51.04 | 0.42 (0.16, 0.71), 0.004**  -0.04 (-0.42, 0.33), 0.81 | 11.19 ± 12.52  -0.40 ± 8.47 | Group ES: -0.31 (-1.17, 0.55), F=0.51, p=0.47  Time ES: 0.57 (-0.16, 1.31), F=2.49, p=0.12  Int ES: -0.61 (-1.83, 0.61), F=1.02, p=0.31 |
| **Relative F_0_ (N.kg^-1^)** | CST  MST | 5.18 ± 0.49  5.10 ± 0.51 | 5.76 ± 0.84  5.07 ± 0.59 | 0.74 (0.22, 1.26), 0.005**  -0.05 (-0.68, 0.57), 0.85 | 11.19 ± 12.52  -0.40 ± 8.47 | Group ES: -0.11 (-0.95,0.73), F=0.07, p=0.78  Time ES: 0.84 (0.12,1.55), F=5.57, p=0.02*  Int ES: -0.88 (-2.06,0.31), F=2.24, p=0.14 |
| **v_0_ (m.s^-1^)** | CST  MST | 8.31 ± 0.83  7.80 ± 0.43 | 8.25 ± 0.60    8.01 ± 0.52 | -0.06 (-0.41, 0.27), 0.69  0.39 (0.04, 0.73), 0.03* | -0.25 ± 6.08  2.60 ± 2.87 | Group ES: -0.77 (-1.66,0.12), F=3.07, p=0.08  Time ES: -0.08 (-0.84,0.67), F=0.04, p=0.82  Int ES: 0.39 (-0.86,1.65) F=0.40, p=0.53 |
| **Absolute P_max_ (W)** | CST  MST | 635.40 ± 144.17  555.32 ± 116.73 | 699.91 ± 175.20  565.94 ± 125.22 | 0.36 (0.13, 0.58), 0.007*  0.08 (-0.18, 0.35), 0.51 | 10.04 ± 11.56  1.94 ± 7.28 | Group ES: -0.52 (-1.38, 0.34), F=0.51, p=0.47  Time ES: 0.42 (-0.31, 1.15), F=2.49, p=0.12  Int ES: -0.35 (-1.57, 0.87), F=1.02, p=0.31 |
| **Relative P_max_ (W.kg^-1^)** | CST  MST | 10.75 ± 1.47  9.96 ± 1.36 | 11.80 ± 1.86  10.15 ± 1.57 | 0.60 (0.18, 1.02), 0.007**  0.12 (-0.27, 0.51), 0.52 | 10.04 ± 11.56  1.94 ± 7.28 | Group ES: -0.46 (-1.30,0.38), F=1.22, p=0.27  Time ES: 0.61 (-0.10,1.33), F=2.99, p=0.09  Int ES: -0.50 (-1.69,0.68), F=0.73, p=0.39 |
